# A Clustering Multi-Criteria Decision-Making Method for Large-Scale Discrete and Continuous Uncertain Evaluation

**DOI:** 10.3390/e24111621

**Published:** 2022-11-08

**Authors:** Siyuan Wang, Wenjun Ma, Jieyu Zhan

**Affiliations:** School of Computer Science, South China Normal University, Guangzhou 510631, China

**Keywords:** multi-criteria decision making, decision making under uncertainty, uncertain information clustering, D–S theory

## Abstract

In recent years, Dempster–Shafer (D–S) theory has been widely used in multi-criteria decision-making (MCDM) problems due to its excellent performance in dealing with discrete ambiguous decision alternative (DA) evaluations. In the general framework of D–S-theory-based MCDM problems, the preference of the DAs for each criterion is regarded as a mass function over the set of DAs based on subjective evaluations. Moreover, the multi-criteria preference aggregation is based on Dempster’s combination rule. Unfortunately, this an idea faces two difficulties in real-world applications: (i) D–S theory can only deal with discrete uncertain evaluations, but is powerless in the face of continuous uncertain evaluations. (ii) The generation of the mass function for each criterion relies on the empirical judgments of experts, making it time-consuming and laborious in terms of the MCDM problem for large-scale DAs. To the best of our knowledge, these two difficulties cannot be addressed with existing D–S-theory-based MCDM methods. To this end, this paper proposes a clustering MCDM method combining D–S theory with the analytic hierarchy process (AHP) and the Silhouette coefficient. By employing the probability distribution and the D–S theory to represent discrete and continuous ambiguous evaluations, respectively, determining the focal element set for the mass function of each criterion through the clustering method, assigning the mass values of each criterion through the AHP method, and aggregating preferences according to Dempster’s combination rule, we show that our method can indeed address these two difficulties in MCDM problems. Finally, an example is given and comparative analyses with related methods are conducted to illustrate our method’s rationality, effectiveness, and efficiency.

## 1. Introduction

MCDM offers a systematic methodology for assisting decision makers in weighing various DAs and determining the optimal DA while considering multiple criteria, which has led to MCDM attracting widespread attention in both theory and practice [1,2]. However, in the practical application of MCDM, uncertainties, such as missing, ambiguous, or inaccurate information, are inevitable due to the subjective evaluations of experts, missing data, the randomness of data, and so on [3,4,5,6]. In order to determine the optimal DA, the MCDM problem in such an uncertain situation usually consists of two major tasks: (1) modeling and processing uncertain information for all evaluations of each decision alternative and (2) synthetically aggregating the performance of each alternative with respect to each criterion [7].

To model and deal with uncertain information, many mathematical tools and theories, such as fuzzy set theory [8], intuitionistic fuzzy set theory [9], rough set theory [10], D–S theory [11,12,13,14,15], evidential reasoning [16], D-number [7], and Z-number [6], have been extended into the uncertain MCDM problem. Among these approaches, as a generalization of probability theory, D–S theory has been found to excel in dealing with uncertain information, such as ambiguous, imprecise, and missing information, due to its main advantages of not requiring additional auxiliary parameters and prior information and having the ability to express uncertainty and ignorance directly [17,18].

Another major task of the uncertain MCDM problem is to synthesize the performance of DAs with respect to each criterion in order to select the optimal DA [7]. A particular number of MCDM techniques have been proposed to be able to accomplish this task, such as the Analytic Hierarchy Process (AHP) [19,20], Technique for Ordering Preference by Similarity to Ideal Solution (TOPSIS) [21], VIsekriterijumska optimizacija i KOmpromisno Resenje (VIKOR) [18], Elimination and Choice Translating Reality (ELECTRE) [22], Best Worst Method (BWM) [23], Measurement of Alternatives and Ranking according to Compromise Solution (MARCOS), and Additive Ratio Assessment (ARAS) [7,13]. Among these techniques, AHP is one of the most representative and widely used [20]. AHP follows the way of thinking of decomposition, comparison, judgment, and synthesis for decision making and establishes a hierarchical structure model, with the top layer as the target layer, the middle layer as the criterion layer, and the bottom layer as the decision alternative layer, which can clearly show the relationships between each layer, each criterion, and each decision alternative [24]. In addition, similarly to the hierarchical structure of the AHP, the D–S theory can also accomplish this task by treating each criterion as a mass function generated by the evaluation of DAs, and the synthesized aggregated evaluation is operationalized by Dempster’s combination rule [25,26]. As the core of D–S theory, Dempster’s combination rule has excellent properties, such as the commutative and associative laws, which enable it to fuse multi-source information efficiently [25,27]. Therefore, D–S theory is widely used in uncertainty modeling and processing [18,28], decision making [29], information fusion [30], etc.

In recent decades, MCDM based on D–S theory has attracted considerable attention and has been extensively studied for its powerful and flexible ability to handle uncertain information and synthesize information from multiple sources [5,13,14,15,18,25,31]. However, there exist two main drawbacks for such D–S theory-based MCDM methods in real-world applications: (i) D–S theory performs well in modeling and handling imprecise, missing, and ambiguous uncertainty evaluations. However, it can only handle discrete uncertainty evaluations and does not handle continuous uncertainty evaluations well [5,13,25]. (ii) Existing methods for generating mass functions for each criterion rely on subjective judgments made with experts’ empirical knowledge, which requires experts to determine the focal element set for the mass function, the mass value of each focal element, or both [5,31]. However, when the number of DAs is huge, the subjective evaluation process is time-consuming and laborious. These two drawbacks set a threshold for the real-world application of D–S-theory-based MCDM methods, and to the best of our knowledge, none of the existing D–S-theory-based MCDM methods can address both drawbacks.

In order to address these drawbacks, this article proposes a clustering MCDM method based on D–S theory, the AHP method, and the Silhouette coefficient. Specifically, we first employ the mass function and probability distribution to represent discrete and continuous ambiguous evaluations on DAs for each criterion. Then, a clustering method based on the Silhouette coefficient is proposed to automatically determine the set of focal elements to generate the mass function of each criterion. Then, to determine the assignment of mass values to each focal element, we propose a new ratio formula for uncertain evaluation values capable of handling both discrete and continuous evaluation values. Since the AHP technique can easily determine the preference of DAs by constructing a judgment matrix and calculating the eigenvectors of the largest eigenvalues, we introduce the eigenvector method of the AHP method to assign the mass value of each focal element. However, the traditional AHP method requires a pairwise comparison of all DAs, which is complicated, while the clustering approach proposed in this paper and the excellent property of the proposed ratio formula can effectively avoid a large number of pairwise comparisons.

Finally, after the mass function of each criterion is discounted according to the importance weight of the criteria, Dempster’s combination rule is applied to merge the mass functions to generate the final evaluation of each DA. Moreover, we confirm the advantages of our method through an example illustration and a comparative analysis with the existing D–S-theory-based MCDM methods.

The contributions of this paper are summarized as follows:

- We identify various continuous uncertain evaluations of DAs in MCDM and represent them by the continuous probability distributions.
- A clustering method based on the Silhouette coefficient is proposed to handle large-scale discrete and continuous uncertain evaluations on DAs in MCDM. Furthermore, when the total number of DAs is relatively large, clustering significantly improves the efficiency of MCDM.
- A new ratio formula for uncertain evaluation values that can handle discrete and continuous uncertain evaluations is proposed. Moreover, we demonstrate that the formula can effectively avoid the large number of pairwise comparisons in the AHP method.
- A clustering MCDM method based on D–S theory and the AHP method is proposed to evaluate all DAs with respect to all related criteria in MCDM.

The rest of this paper is structured as follows: The studied literature is outlined in Section 2; Section 3 reviews the basic concepts of D–S theory and other preliminaries used in this paper; a novel MCDM method is proposed in Section 4; Section 5 details the experimental evaluations; Section 6 concludes this paper.

## 2. Literature Review

Solving the uncertain MCDM problem consists of two crucial tasks: the first is modeling and processing all of the uncertain information, and the second is integrating the performance of DAs under each criterion to determine the optimal DA [7].

In order to achieve the first task, some theories and methods for dealing with uncertain information have been incorporated into MCDM problems. For example, the fuzzy set theory provides a systematic framework and solves uncertainty and fuzzy problems with the aid of the membership function [8]. Then, the intuitionistic fuzzy set was proposed, which extended fuzzy sets to represent the rejection degree by the non-membership degree [9]. Then, an interval-valued intuitionistic fuzzy set was proposed, where both the membership and non-membership degree are represented by intervals [32]. To represent the reliability of the information, the Z-number was then proposed [6]. Similarly, the existing extended fuzzy set theories, such as spherical fuzzy sets and neutrosophic sets, additionally considered the mental state of the decision maker, especially the hesitation state. However, it is too complicated for the decision maker to define their satisfaction, dissatisfaction, and hesitation level when expressing thoughts [33,34]. Furthermore, rough set theory was proposed to solve the inconsistency problem of granular information [10], and soft set theory provided a general mechanism for modeling uncertainty from a parametric point of view [35]. The D-number was proposed to express imprecision and uncertainty [7]. The evidential reasoning rule presented a general approach to solving uncertain information, including ignorance and randomness [16]. As an extension of probability theory, the D–S theory was proposed by Dempster and developed by Shafer. The mass function in the D–S theory can express imprecision, ambiguity, and ignorance by assigning support to subsets [11,12]. The D–S theory performs very well in modeling and handling uncertain information without additional auxiliary information, such as the membership function in fuzzy set theory, the non-membership function in intuitionistic fuzzy set theory, and the reliability in the Z-number [13,14,15,18,28].

The second crucial task of the uncertain MCDM problem is to combine the performance of all DAs for each criterion and obtain the optimal DA. A considerable number of traditional MCDM technologies can do this, such as AHP [19,20], TOPSIS [21], VIKOR [18], BWM [23], ELECTRE [22], MARCOS, and ARAS [7,13]. In addition to these traditional MCDM techniques, the D–S theory can also be used to select the optimal DA by treating each criterion as a mass function whose frame of discernment is the set of all DAs and then combining the mass functions of all the criteria by Dempster’s combination rule [25,26]. Furthermore, Dempster’s combination rule satisfies the commutative and associative laws, making it very efficient in fusing information from multiple sources [11,12].

D–S theory performs well in modeling and handling multiple types of uncertain information, and its core, Dempster’s combination rule, can efficiently fuse information from multiple criteria [13,14,15,18,28]. Therefore, incorporating D–S theory into the uncertain MCDM problem is desirable and has attracted much attention from researchers in recent years. For example, the EFMCDM model proposed by Xiao et al. combines confidence entropy and Dempster’s combination rule [25]; Zhou et al. put forward the method of using the evidential reasoning rule to replace Dempster’s combination rule in MCDM to make decisions [36]; Beynon et al. proposed a DS/AHP method that combines D–S theory with the AHP method, with which decision makers can make preferential judgments on the group of DAs instead of comparing individual DAs or making pairwise comparison of DAs [37]; Hua et al. proposed the DS-AHP method to deal with incomplete information [27]; Ma et al. identified various types of ambiguous uncertain information and extended the DS/AHP method to deal with such uncertain information [5]; Chattopadhyay et al. proposed the use of the D-number, an extended version of D–S theory, to deal with uncertain information and select suppliers in the steel industry with the help of the MARCOS method [7]; Fei et al. fused D–S theory with the ELECTRE and VIKOR methods to select optimal suppliers in supply chain management [15,18]; Hatefi et al. fused D–S theory and ARAS methods for sustainable construction material selection [13]; Wang et al. used the D–S theory to represent and handle uncertain information and used TOPSIS to select the optimal offshore wind turbine [14].

However, these existing methods based on the D–S theory rely on the empirical knowledge of experts when generating the mass function of each criterion, which is time-consuming, labor-intensive, and subjective. Moreover, these methods can only deal with discrete evaluations and not continuous evaluation values, which are very common in the practical applications of MCDM. Therefore, to tackle the above problems, a novel MCDM method is proposed in this paper.

## 3. Preliminaries

First, we review the basic definitions in D–S theory [11].

**Definition 1.**  *Let $\Theta =\{\theta_{1},\ldots ,\theta_{n}\}$ be a set of exhaustive and mutually exclusive elements called a frame of discernment (or, simply, a frame). A function $m\;:\;2^{\Theta }\;\to \;\left[0,1\right]$ is a mass function over *Θ* if*

$$m\left(\emptyset \right)=0,\;\sum_{A\subseteq \Theta }m\left(A\right)=1.$$

*A function $Pl:2^{\Theta }\to \left[0,1\right]$ is a plausibility function if*

$$Pl\left(A\right)=\sum_{B\cap A\neq \phi }m\left(B\right).$$

*Here, the mass value of m(A) represents the degree to which the corresponding evidence supports A. Moreover, any subset A⊆Θ satisfying m(A)>0 is called a focal element of m. In particular, the mass function of m(Θ)=1 represents the decision maker’s total ignorance over the frame *Θ*.*

One advantage of D–S theory is that it provides a method for accumulating and combining evidence from multiple distinct sources by using Dempster’s combination rule [12].

**Definition 2.**  *(Dempster’s combination rule) Let $m_{1}$ and $m_{2}$ be two mass functions over a frame of discernment* Θ. *Then, the combined mass function from $m_{1}$ and $m_{2}$ according to Dempster’s combination rule, denoted as $m_{1,2}$, is defined as: *

(1) $$m_{1,2}\left(x\right)=\left\{\begin{array}{cc} 0 & ifx=\emptyset ,\\ \frac{\sum_{A⋂B=x}m_{1}\left(A\right)m_{2}\left(B\right)}{1-\sum_{A⋂B=\emptyset }m_{1}\left(A\right)m_{2}\left(B\right)}\; & ifx\neq \emptyset . \end{array}\right.$$

Since the probability of identifying each element in the frame is uncertain for the mass function, some methods are employed to transform uncertain probability into certain probability, which is called probability transformation [38].

**Definition 3.**  *(Pignistic probability function) Let m be a mass function over *Θ. *Its associated pignistic probability function $BetP_{m}:\Theta \to \left[0,1\right]$ is defined as:*

(2) $$\begin{array}{c} BetP_{m}\left(\theta \right)=\sum_{A\subseteq \Theta ,\theta \in A}\frac{1}{\left|A\right|}\frac{m(A)}{1-m(\emptyset )},m\left(\emptyset \right)<1. \end{array}$$

*where |A| is the cardinality of A.*

Due to the existence of the criterion weight, it is necessary to discount the criterion’s mass function according to the weight before combining the mass functions of all criteria. Shafer’s discounting method is defined as follows [12].

**Definition 4.**  *(Shafer’s discounting) Let m be a mass function over a frame *Θ*, and let* ω *be the evidence weight of mass function m; then, the mass function $\hat{m}$ discounted by the evidence weight is given by:*

(3) $$\begin{array}{c} \hat{m}\left(A\right)=\left\{\begin{array}{cc} 0 & \;\mathrm{if}\;A=\emptyset ,\\ \omega \cdot m(A) & \;\mathrm{if}\;A\subset \Theta \;\mathrm{and}\;A\neq \emptyset ,\\ \omega \cdot m(A)+(1-\omega ) & \;\mathrm{if}\;A=\Theta . \end{array}\right. \end{array}$$

Based on the concept of D–S theory, Ma et al. [4] proposed four types of discrete ambiguous evaluation for DAs with respect to a given criterion and converted them into the form of mass function as follows.

**Definition 5.**  *(Discrete ambiguous evaluation) Let $m_{A,c}$ be the mass function representing the decision maker’s judgment about the DA, A, regarding criterion c, and let $H=\{x_{i}|x_{i}\in R,i=1,\ldots ,n,x_{1}<\ldots <x_{n}\}$ be a mutually exclusive and collectively exhaustive set of numeral assessments that present a scale set of n-scale unit preference on DAs; then, the four forms of evaluation and their corresponding mass functions are defined as follows:*

- *Determined evaluation.*
*This can be expressed as a special mass function defined on the frame H: $m_{A,c}\left(\left\{x\right\}\right)=1$, where x(x∈H) is the numeral assessment grade evaluated by an expert.*
- *Unknown evaluation.*
  *Since the decision maker is utterly ignorant in this situation, it can be expressed as a special mass function defined on the frame* Θ*: $m_{A,c}\left(\Theta \right)=1$, Θ=H.*
- *Interval-valued evaluation.*
  *This means that the decision maker only knows that the numeral assessment grade could be anywhere between $x_{i}$ and $x_{j}$, where $x_{i}$ and $x_{j}$ are the numeral assessment grades evaluated by the expert, but it is not sure which one. It can be expressed as a special mass function defined on the frame* Θ*: $m_{A,c}\left(\left\{x_{i},\cdots ,x_{j}\right\}\right)=1$, Θ=H.*
- *Ambiguous evaluation.*
  *This can be expressed as a special mass function defined on the frame* Θ*: $m_{A,c}$, where $\sum_{D\subseteq \Theta }m_{A,c}\left(D\right)=1$, Θ=H, and $\exists T\subseteq H,|T|\geq 1,m_{A,c}\left(T\right)>0$.*

The Silhouette coefficient is an index for judging the clustering effect [39] among a set of elements. The closer the distance between samples of the same class and the farther the distance between samples of different classes, the better the clustering effect and the larger the Silhouette coefficient.

**Definition 6.**  *(Silhouette coefficient) Let $X=\{x_{1},\ldots ,x_{n}\}$ be a set of elements, let $\left\{Cl_{1},\ldots ,Cl_{k}\right\}$, and let (k<n) be a set of clusters, where $Cl_{i}$ is the cluster consisting of $x_{i}$; then, the Silhouette coefficient of the k clusters is given by:*

(4) $$\begin{array}{c} SC\left(k\right)=\frac{1}{n}\sum_{i=1}^{n}\frac{b\left(x_{i}\right)-a\left(x_{i}\right)}{\max\{a\left(x_{i}\right),b\left(x_{i}\right)\}}, \end{array}$$

*where $a(x_{i})$ is the average distance between $x_{i}$ and other data in the cluster $Cl_{i}$, and $b(x_{i})$ is the minimum value of the average distance between $x_{i}$ and another cluster $Cl_{t}\left(1\leq t\leq k,t\neq i\right)$, given by:*

(5) $$\begin{array}{c} a\left(x_{i}\right)=\frac{\sum_{j\in Cl_{i},j\neq i}\eta \left(x_{i},x_{j}\right)}{|Cl_{i}|}, \end{array}$$

(6) $$\begin{array}{c} b\left(x_{i}\right)=\min_{1\leq t\leq k,t\neq i}\frac{\sum_{j\in Cl_{t}}\eta \left(x_{i},x_{j}\right)}{|Cl_{t}|}, \end{array}$$

*in which $\eta (x_{i},x_{j})$ is the distance between $x_{i}$ and $x_{j}$.*

## 4. A Novel MCDM Method

Based on the D–S theory, clustering method, and eigenvector method in the AHP, this section proposes a novel MCDM method for dealing with uncertain information.

*Problem definition*: Let $\Lambda =\left\{A_{1},A_{2},\ldots ,A_{n}\right\}$ be the nonempty finite DA set consisting of *n* DAs, let $C=\left\{c_{1},c_{2},\ldots ,c_{m}\right\}$ be the nonempty finite decision criteria set, let $x_{l,i}\;\left(1\leq l\leq n,1\leq i\leq m\right)$ be the value (certain or uncertain) of the DA $A_{l}\;\left(A_{l}\in \Lambda \right)$ regarding the criterion $c_{i}\;\left(c_{i}\in C\right)$, and let $\omega_{i}$ be the importance weights of the decision criterion $c_{i}\;\left(i=1,2,\ldots ,m\right)$. For this uncertain MCDM problem, the optimal DA should be selected from Λ.

For example, an automotive enterprise needs to select an optimal DA from ten suppliers of automotive parts, and these ten suppliers are denoted as *a*, *b*, *c*, *d*, *e*, *f*, *g*, *h*, *i*, and *j*, respectively. In this MCDM problem, automotive enterprises evaluate suppliers using four decision criteria: price, delivery time, quality, and service level. The evaluations for DAs over all criteria are shown in Table 1, where some values are unknown (denoted as “null”) due to the absence of data values, and P(·) refers to the probability. For the criteria of service level and quality, the grades of DAs could be evaluated by the following linguistic terms: Very Poor (VP), Poor (P), Average (A), Good (G), and Very Good (VG). We can find that some evaluations of DAs are discrete uncertain values and some evaluations of DAs are continuous uncertain values. Moreover, such MCDM problems are common in real-world applications, and existing methods cannot solve them.

**Figure 1:**
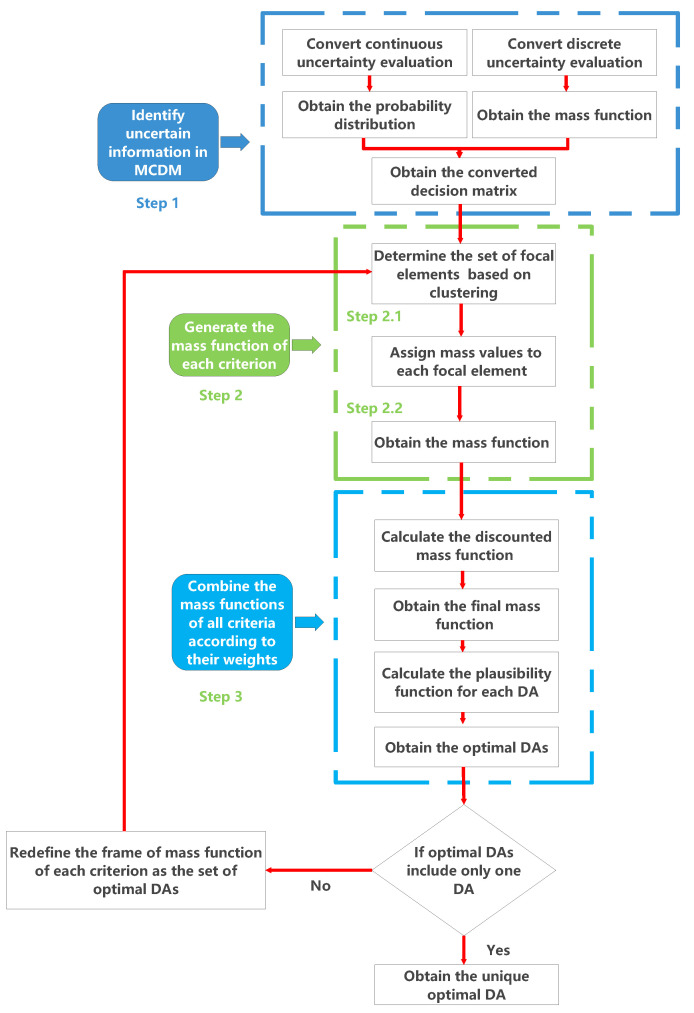
Flowchart of the proposed method.

In order to solve the MCDM problem shown in Table 1 and select the optimal DA, the proposed method is shown in Figure 1. As shown in the figure, the proposed method has three main steps, including identifying the uncertain information in the MCDM, generating the mass function of each criterion, and combining the mass functions of all criteria according to their weights. The specific steps are shown below.

### 4.1. Step 1: Identifying Uncertain Information in MCDM

In Section 2, we recall several forms of discrete evaluation values for DAs identified by Ma et al. [4]. Then, inspired by this idea, we identify the various forms of continuous evaluation values of DAs as follows.

For a continuous evaluation value $x_{l,i}$ of a DA $A_{l}\left(A_{l}\in \Lambda \right)$ regarding a criterion $c_{i}\left(c_{i}\in C\right)$, if Z(Z⊆R) is the region in which the evaluation value is defined, $x_{l,i}$ could take one of the following three forms:

- Missing evaluation value, such as the delivery time of supplier *i* in Table 1.
- Interval value: $[a,b],$ where a,b∈Z and a<b, such as the price of supplier *a* in Table 1.
- Continuous probability distribution: $\int_{a}^{b}f_{A,c}\left(x\right)dx=1$, where a,b∈Z,a<b and $f_{A,c}\left(\cdot \right)$ is the probability density function, such as the delivery time of supplier *a* in Table 1.

In order to facilitate the processing of evaluation values, we convert all evaluation values into continuous probability distributions, as shown below.

- Since we are entirely ignorant of missing evaluations, we assume that it takes a random value in the interval and obeys a uniform distribution. Furthermore, the lower bound of the interval is assumed to be the minimum possible value (the worst case) of all other DAs, while the upper bound of the interval is assumed to be the maximum possible value (the best case) of all other DAs. Moreover, such assumptions satisfy our intuition that in many real-world applications, the worst case of missing values is no worse than the known worst case, and the best case is no better than the known best case.
- For the case of interval evaluation, since its probability distribution is unknown, generally, interval-valued evaluation is uniformly treated along its range [40]. Then, we can express it as a uniform probability distribution, $\int_{a}^{b}f_{A,c}\left(x\right)dx=1$, where a,b∈Z,a<b, which is a continuous probability distribution with a constant probability density function $\frac{1}{b-a}$.

Therefore, all forms of discrete evaluation values of DAs can be expressed in the form of mass functions, while all forms of continuous evaluation values of DAs can be expressed in the form of probability distributions.

### 4.2. Step 2: Generating the Mass Function of Each Criterion

In this paper, the generation of the mass function of each criterion consists of two steps: Firstly, DAs are divided into different clusters according to their values using the clustering method, where the set of DAs divided into the same cluster constitutes a focal element of the mass function; secondly, the preference of each cluster, which is the mass value of each focal element, is calculated to generate the mass function of the criterion.

#### 4.2.1. Step 2-1: Determining the Set of Focal Elements Based on Clustering

In this paper, we update the UK-medoids, one of the classic methods for clustering uncertain data [41], in order to cluster the *n* DAs, and we use the Silhouette coefficient in Definition 6 to determine the optimal number of clusters and the set of clusters for the DAs.

Firstly, the distance between values in the clustering algorithm needs to be calculated. We define the distance between uncertainty values in the form of a probability distribution and mass function as follows.

**Definition 7.**  *(Distance of uncertain values) Let $x_{i}$ be the value, let $Z_{i}$ be the region in which value $x_{i}$ is defined, let $f_{i}\left(\cdot \right)$ be the probability of $x_{i}$ if $x_{i}$ is in the form of a continuous probability distribution, let $BetP_{m_{i}}\left(\cdot \right)$ be the pignistic probability of $m_{i}$, and let* Θ *be the frame of $m_{i}$ if $x_{i}$ is in the form of a mass function $m_{i}$; then, the distance between two values $x_{i}$ and $x_{j}$, denoted as $\eta \left(x_{i},x_{j}\right)$, is defined by the following:*

- *If $x_{i}$ and $x_{j}$ are both continuous probability distributions:*
  $$\eta \left(x_{i},x_{j}\right)=\int_{x\in Z_{i}}\int_{y\in Z_{j}}\left|x-y\right|\cdot f_{i}\left(x\right)\cdot f_{j}\left(y\right)dxdy;$$
- *If $x_{i}$ and $x_{j}$ are both mass functions:*
  $$\eta \left(x_{i},x_{j}\right)=\sum_{\theta_{i}\in \Theta }\sum_{\theta_{j}\in \Theta }\left|\theta_{i}-\theta_{j}\right|\cdot BetP_{m_{i}}\left(\theta_{i}\right)\cdot BetP_{m_{j}}\left(\theta_{j}\right);$$
- *If $x_{i}$ is a mass function and $x_{j}$ is a continuous probability distribution:*
  $$\eta \left(x_{i},x_{j}\right)=\sum_{\theta_{i}\in \Theta }\cdot BetP_{m_{i}}\left(\theta_{i}\right)\cdot \int_{y\in Z_{j}}\left|\theta_{i}-y\right|\cdot f_{j}\left(y\right)dy.$$

Then, our proposed clustering algorithm is shown in Algorithm 1.

**Algorithm1:** Clustering of values of DAs.

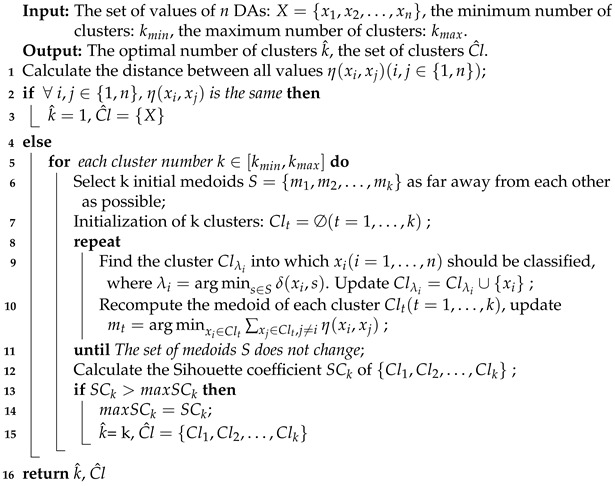



Overview. Given the set of values of each DA and the predetermined minimum and maximum numbers of clusters, $k_{min}$ and $k_{max}\left(2\leq k_{min}\leq k_{max}\leq n\right)$, Algorithm 1 can determine the optimal number of clusters and obtain the set of clusters. The parameters $k_{min}$ and $k_{max}$ are two predetermined parameters that indicate the lower and upper limits of the number of clusters taken, respectively, so that the optimal number of clusters is between the values of these two parameters. Moreover, $k_{min}$ and $k_{max}$ can be the same, and when they are the same, it is equivalent to the decision maker fixing the number of clusters to a single value. The main procedures are as follows: (1) First, calculate the distance between all values of the DAs, including the distance between the value and itself. Note that if the value of the DA is uncertain, the distance between the value of DA and itself is greater than 0. (2) If all of the distances calculated above are the same, then all values of DAs regarding this criterion are considered the same and should be divided into the same cluster (shown in lines 2–3 of Algorithm 1), in which case the mass function generated by this criterion represents ignorance in D–S theory. Otherwise, all values of DAs will be clustered based on the distance between values. The number of clusters is in the range of $[k_{min},k_{max}]$, so the number of clusters whose Silhouette coefficient reaches the maximum is the optimal number of clusters, and the set of clusters obtained at this time is the optimal set of clusters (shown in lines 5–15 of Algorithm 1). In addition, if the set of clusters derived from Algorithm 1 contains empty clusters, then the empty clusters are not added to the set of optimal clusters, so the set of optimal clusters contains only clusters with elements.

Compared with the UK-medoids algorithm, Algorithm 1 has the following advantages: (1) The formula for calculating the distance of uncertain values is extended to apply to the uncertain values in the form of the mass function, as shown in Definition 7. (2) The number of clusters can be automatically determined according to the Silhouette coefficient without determining the number of clusters in advance. Note that if all distances are the same, the number of clusters is 1. That is, all values are divided into the same cluster. In this case, all DAs with respect to this criterion behave the same, so it is only through this criterion that DAs cannot be distinguished. Therefore, all DAs should be grouped into the same cluster, in which case the mass function generated by this criterion represents ignorance in D–S theory. Therefore, when all values are the same, it is unnecessary to divide them into two or more clusters. (3) The selection method of the initial clustering medoid is improved. Unlike in the random selection of cluster centers, the improved selection method can maximize the distance between the initial medoids so that the different final clusters are as far from each other as possible. The specific steps are as follows: First, randomly select a value as the first initial medoid, select the value with the largest distance from the first initial medoid as the second initial medoid, and then select the value with the largest minimum distance from the first two values as the third initial medoid, and so on, until *k* initial medoids are selected.

Then, by applying Algorithm 1, we will obtain the optimal number of clusters, denoted as $\hat{k}$, and the set of clusters $\left\{Cl_{1,i},Cl_{2,i},\ldots ,Cl_{\hat{k},i}\right\}$ regarding the criterion $c_{i}$. The set of optimal clusters determines the set of focal elements of the mass function of the criterion, where all DAs contained in each cluster constitute a focal element.

#### 4.2.2. Step 2-2: Assigning Mass Values to Each Focal Element

After obtaining the set of clusters corresponding to the criterion, we need to determine each cluster’s preference degree to generate the criterion’s mass function. It should be noted that if the number of clusters regarding the criterion $c_{i}\left(c_{i}\in C\right)$ obtained by Algorithm 1 is 1, that is, all DAs regarding criterion $c_{i}$ have the same performance, this means that the pros and cons of each DA cannot be distinguished regarding criterion $c_{i}$. The information provided by this criterion is completely ignorant, so the mass function of criterion $c_{i}$ is $m_{c_{i}}\left(\Theta \right)=1$. The following content of this subsection presents the case where the number of clusters is greater than or equal to 2.

In this paper, we determine the preference degree of each cluster by calculating the ratio relationship of the values among different clusters. Therefore, before calculating the preference degree of each cluster, the ratio between all the values contained in the different clusters needs to be calculated. Similarly to the distance of uncertain values, the formula for calculating the ratio between uncertain values is as follows.

**Definition 8.**  *(Ratio of uncertain values) Let $x_{i}$ be the value, let $Z_{i}$ be the region in which value $x_{i}$ is defined, let $f_{i}\left(\cdot \right)$ be the probability of $x_{i}$ if $x_{i}$ is in the form of probability distribution, let $BetP_{m_{i}}\left(\cdot \right)$ be the pignistic probability of $m_{i}$, and let* Θ *be the frame of $m_{i}$ if $x_{i}$ is in the form of a mass function. Then, the ratio of value $x_{i}$ to $x_{j}$, denoted as $r\left(x_{i},x_{j}\right)$, is defined by the following:*

- *If $x_{i}$ and $x_{j}$ are both continuous probability distributions:*
  $$r\left(x_{i},x_{j}\right)=\int_{x\in Z_{i}}\int_{y\in Z_{j}}\left(x/y\right)\cdot f_{i}\left(x\right)f_{j}\left(y\right)dxdy;$$
- *If $x_{i}$ and $x_{j}$ are both mass functions:*
  $$r\left(x_{i},x_{j}\right)=\sum_{\theta_{i}\in \Theta }\sum_{\theta_{j}\in \Theta }\left(\theta_{i}/\theta_{j}\right)\cdot BetP_{m_{i}}\left(\theta_{i}\right)\cdot BetP_{m_{j}}\left(\theta_{j}\right);$$
- *If $x_{i}$ is a mass function and $x_{j}$ is a continuous probability distribution:*
  $$r\left(x_{i},x_{j}\right)=\sum_{\theta_{i}\in \Theta }BetP_{m_{i}}\left(\theta_{i}\right)\cdot \int_{y\in Z_{j}}\left(\theta_{i}/y\right)\cdot f_{j}\left(y\right)dy.$$

The proposed formula for the ratio of uncertain values has the following excellent property.

**Theorem 1.**  *Let $r\left(x_{i},x_{j}\right)$ be the ratio of $x_{i}$ to $x_{j}$, let $r\left(x_{i},x_{k}\right)$ be the ratio of $x_{i}$ to $x_{k}$, let $r\left(x_{j},x_{j}\right)$ be the ratio of $x_{j}$ to itself, and let $Z_{i}$ be the region in which value $x_{i}$ is defined; then, the ratio of $x_{j}$ to $x_{k}$, denoted as $r\left(x_{j},x_{k}\right)$, can be expressed in terms of $r\left(x_{i},x_{j}\right)$, $r\left(x_{i},x_{k}\right)$, and $r\left(x_{j},x_{j}\right)$ as follows.*

$$r\left(x_{j},x_{k}\right)=\frac{r\left(x_{j},x_{j}\right)\cdot r\left(x_{i},x_{k}\right)}{r\left(x_{i},x_{j}\right)}$$

**Proof.**  Considering the case of the continuous probability distribution, according to Fubini’s theorem [42], when the integral area of a double integral is a rectangular area and the binary function can be separated into the product of two univariate functions, the double integral can be converted to a repeated integral. Thus, we have

$$r\left(x_{i},x_{j}\right)=\int_{x\in Z_{i}}x\cdot f_{i}\left(x\right)dx\cdot \int_{y\in Z_{j}}\left(1/y\right)\cdot f_{j}\left(y\right)dy;\;r\left(x_{i},x_{k}\right)=\int_{x\in Z_{i}}x\cdot f_{i}\left(x\right)dx\cdot \int_{z\in Z_{k}}\left(1/z\right)\cdot f_{k}\left(z\right)dz;\;r\left(x_{j},x_{k}\right)=\int_{y\in Z_{j}}y\cdot f_{j}\left(y\right)dy\cdot \int_{z\in Z_{k}}\left(1/z\right)\cdot f_{k}\left(z\right)dz.$$

Then, we have

$$\begin{array}{cc} \; & r\left(x_{i},x_{j}\right)\cdot r\left(x_{j},x_{k}\right)\\ \; & =\int_{x\in Z_{i}}x\cdot f_{i}\left(x\right)dx\cdot \int_{y\in Z_{j}}\left(1/y\right)\cdot f_{j}\left(y\right)dy\cdot \int_{x\in Z_{i}}x\cdot f_{i}\left(x\right)dx\cdot \int_{z\in Z_{k}}\left(1/z\right)\cdot f_{k}\left(z\right)dz\\ \; & =r\left(x_{j},x_{j}\right)\cdot r\left(x_{i},x_{k}\right) \end{array}$$

In the case of discrete forms, the integral needs to be transformed into a cumulative form, and the procedure of the proof is similar. Thus, Theorem 1 holds. □

Therefore, by Theorem 1, we only need to calculate the ratio of each uncertain value to itself and the ratio of one uncertain value to all other uncertain values; then, the ratio between all uncertain values can be calculated, which will significantly reduce the number of comparisons and calculations and improve the efficiency.

Next, we can obtain the ratio relationship between different clusters, as shown below.

**Definition 9.**  *(Ratio of clusters) Let $r(x_{i},x_{j})$ be the ratio of uncertain value $x_{i}$ to $x_{j}$; then, the ratio of cluster $Cl_{h,i}$ to $Cl_{t,i}$ regarding the criterion $c_{i}\left(c_{i}\in C\right)$, denoted as $R_{i}\left(h,t\right)$, is defined by:*

$$R_{i}\left(h,t\right)={\left(\prod_{\begin{array}{c} x_{i}\in Cl_{h,i},x_{j}\in Cl_{t,i} \end{array}}r\left(x_{i},x_{j}\right)\right)}^{\frac{1}{|Cl_{h,i}|\cdot |Cl_{t,i}|}}$$

*where $|Cl_{h,i}|,|Cl_{t,i}|$ are the numbers of evaluations contained in clusters $Cl_{h,i}$ and $Cl_{t,i}$.*

It is important to note that the ratio between uncertain values of the same cluster does not need to be calculated. Moreover, due to Theorem 1, only the ratio of one uncertain value to other values needs to be calculated; then, the ratios of all uncertain values can be obtained. Therefore, we need to pick the cluster that contains the largest number of uncertain values, randomly pick one of the uncertain values, and then calculate the ratio between it and all the values of other clusters. The ratio of all uncertain values can be obtained; thus, the ratio between the clusters can also be obtained. Therefore, the efficiency of calculating the ratio between all clusters is significantly improved by Theorem 1.

After the ratio relationships of all clusters are obtained, we use the AHP method to determine the preference degree of each cluster regarding the criterion. First, we construct the judgment matrix for pairwise comparisons among clusters regarding the criterion $c_{i}$. Let $R_{i}\left(h,t\right)$ be the ratio of cluster $Cl_{h,i}$ to $Cl_{t,i}$ and let *k* be the number of clusters; then, the judgment matrix is constructed as follows.

- If $c_{i}$ is a benefit criterion:
  $$[\begin{array}{cccc} R_{i}\left(1,1\right) & R_{i}\left(1,2\right) & \ldots & R_{i}\left(1,k\right)\\ R_{i}\left(2,1\right) & R_{i}\left(2,2\right) & \ldots & R_{i}\left(2,k\right)\\ \vdots & \ldots & \ldots & \vdots \\ R_{i}\left(k,1\right) & R_{i}\left(k,2\right) & \ldots & R_{i}\left(k,k\right) \end{array}]$$
- If $c_{i}$ is a cost criterion:
  $$[\begin{array}{cccc} 1/R_{i}\left(1,1\right) & 1/R_{i}\left(1,2\right) & \ldots & 1/R_{i}\left(1,k\right)\\ 1/R_{i}\left(2,1\right) & 1/R_{i}\left(2,2\right) & \ldots & 1/R_{i}\left(2,k\right)\\ \vdots & \ldots & \ldots & \vdots \\ 1/R_{i}\left(k,1\right) & 1/R_{i}\left(k,2\right) & \ldots & 1/R_{i}\left(k,k\right) \end{array}]$$

When the criterion is a benefit criterion, the elements of the judgment matrix are the ratio of each cluster. When the criterion is a cost criterion, the elements are the reciprocal of the ratio of each cluster. For the positive reciprocal matrix, the maximum eigenvalue must be a positive eigenvalue according to the Perron–Frobenius theorem [43], and its corresponding eigenvector is a positive vector. The eigenvector is then normalized, and the normalized eigenvector of the maximum eigenvalue can be used as the weight vector. Therefore, the normalized eigenvector corresponding to the largest eigenvalue of the judgment matrix is selected as the weight vector, in which each element value is the preference degree of each cluster Cl, denoted as μ(Cl). Then, we can obtain the mass function of criterion $c_{i}$ as follows.

**Definition 10.**  *(The mass function of the criteria) Let $\left\{Cl_{1,i},Cl_{2,i},\ldots ,Cl_{k,i}\right\}$ be the set of k clusters regarding the criterion $c_{i}\left(c_{i}\in C\right)$, let $\mu (Cl_{t,i})$ be the preference degree of the cluster $Cl_{t,i}$, and let $U_{t,i}$ be the union set of DAs corresponding to all values contained in $Cl_{t,i}$, where $1\leq t\leq \hat{k}$ and $U_{t,i}\subseteq \Lambda$; then, the mass function of criterion $c_{i}$ defined on the frame* Λ*, denoted by $m_{c_{i}}$, is given by*

$$m_{c_{i}}\left(U_{t,i}\right)=\frac{\mu (Cl_{t,i})}{\sum_{j=1}^{\hat{k}}\mu \left(Cl_{j,i}\right)}\;and\;\sum_{t=1}^{\hat{k}}m_{c_{i}}\left(U_{t,i}\right)=1.$$

### 4.3. Step 3: Combining the Mass Functions of All Criteria According to Their Weights

After obtaining the mass functions of all criteria in Step 2, by Definition 4, we can obtain the mass function of each criterion $c_{i}\left(c_{i}\in C\right)$ discounted by the importance weight $\omega_{i}$, which is denoted as ${\hat{m}}_{i}$. Then, through Dempster’s combination rule in Definition 2, we combine all of the discounted mass functions of each criterion to obtain the final mass function defined on the frame Λ, denoted as $m_{final}$. After obtaining the final mass function, according to Definition 1, we calculate the plausibility function of each DA within the frame of the final mass function. Then, the optimal DAs are the DAs with the maximum plausibility function. In this step, if two or more DAs have the same maximum plausibility function (that is, they are divided into the same cluster with regard to each criterion), we can redefine the frame of the mass function of each criterion as the set of DAs to be further compared and repeat Steps 2–3 until we obtain the unique optimal DA.

## 5. Experiments

**Figure 2:**
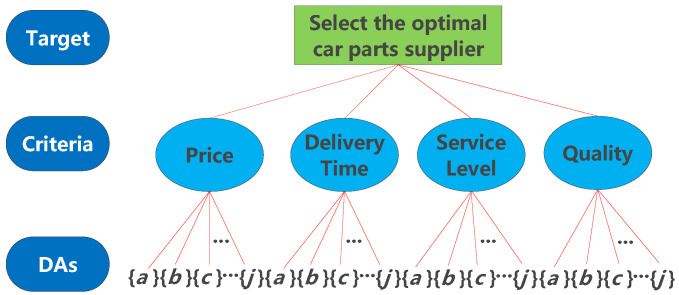
Decision tree for the selection of a car part supplier.

In this section, we will first illustrate the novel MCDM method proposed in this paper using the decision-making problem of selecting the optimal supplier of car parts described in Section 3, as shown in Table 1. Moreover, the decision tree following the three-level AHP framework is shown in Figure 2. Then, a comparative analysis of our method and the existing related MCDM methods is performed.

### 5.1. Illustration of the Proposed Method

#### 5.1.1. Step 1: Identifying Uncertain Information in MCDM

For qualitative criteria, in this paper, the set *H* of numeral assessment grades we use is $\left\{1,2,3,\ldots ,9\right\}$, which represents a nine-scale unit preference set for DAs, and the corresponding numeral assessment grades of the linguistic terms Very Poor (VP), Poor (P), Average (A), Good (G), and Very Good (VG) are 1,3,5,7, and 9, respectively, as shown in Table 2. We convert the continuous uncertain evaluations and discrete uncertain evaluations in Table 1 into the form of probability distributions or mass functions, as shown in Table 3.

#### 5.1.2. Step 2: Generating the Mass Function of Each Criterion

In Step 2, we first generate the set of focal elements of the mass function for each criterion by clustering; then, we assign mass values to each focal element.

##### Step 2-1: Determining the Set of Focal Elements Based on Clustering

By setting the minimum and the maximum numbers of clusters as 2 and 10, respectively, and applying Algorithm 1, we can obtain the optimal set of clusters of DAs for each criterion, which determines the set of focal elements of the mass function of the criterion, where all DAs contained in each cluster form a focal element. The optimal set of clusters of DAs is shown in Table 4.

**Figure 3:**
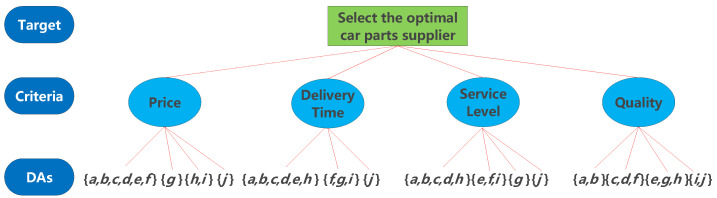
Decision tree of car part suppliers after clustering.

After determining the set of focal elements based on clustering, a new decision tree for the selection of a car part supplier is shown in Figure 3.

##### Step 2-2: Assigning Mass Values to Each Focal Element

After obtaining the optimal set of clusters of the criterion, we choose the largest cluster among the set of clusters of the criterion; then, we choose a random value in that cluster and calculate the ratio between it and all values of other clusters according to Definition 8. The ratio between all values can be obtained according to Theorem 1. Then, according to Definition 9, the ratio between all clusters can be obtained. We construct the judgment matrix and calculate the preference degree of each cluster with the eigenvector method. Then, according to Definition 10, the mass function of this criterion is obtained, as shown in Table 5.

#### 5.1.3. Step 3: Combining the Mass Functions of All Criteria According to Their Weights

In this article, we weight the criteria of price, delivery time, service level, and quality as $\omega (m_{p})=0.4$, $\omega (m_{t})=0.1$, $\omega (m_{sl})=0.2$, and $\omega (m_{q})=0.3$, respectively. Then, we obtain the discounted mass function of each criterion with Definition 4, as shown in Table 6.

By using Dempster’s combination rule, we can obtain the final mass function, denoted as $m_{final}$, as shown in Table 7.

Then, according to Definition 1, we can obtain the plausibility function of each DA as follows:

$$\begin{array}{c} Pl(a)=Pl(b)=0.546,Pl(c)=Pl(d)=0.562,\\ Pl(e)=0.553,Pl(f)=0.505,Pl(g)=0.504,\\ Pl(h)=0.535,Pl(i)=0.496,Pl(j)=0.485. \end{array}$$

Therefore, based on the values of the plausibility functions of all of the DAs, we can derive the priority order of all of the DAs as c,d≻a,b≻e≻h≻f≻g≻i≻j. It can be seen that the DAs *c* and *d* are the two optimal alternatives among them. Furthermore, if we want to determine the unique optimal DA, we need to further compare DAs *c* and *d*. We redefine the frame of the mass function of each criterion as $\Theta^{\prime }=\left\{c,d\right\}$ and repeat Steps 2–3; then, we can obtain the final combined mass function as follows:

$$\begin{array}{cc} \; & m_{final}^{\Theta^{\prime }}\left(\left\{c\right\}\right)=0.24,m_{final}^{\Theta^{\prime }}\left(\left\{d\right\}\right)=0.21,\\ \; & m_{final}^{\Theta^{\prime }}\left(\Theta^{\prime }\right)=0.55. \end{array}$$

Then, we can obtain the plausibility functions of DAs *c* and *d* as: Pl(c)=0.79,Pl(d)=0.76. Therefore, c≻d, and the optimal DA is *c*.

### 5.2. Comparison with Related Work and Discussion

We first compare our proposed MCDM method with existing MCDM methods based on D–S theory, including DS/AHP [37], DS-AHP [27], Ma et al.’s method [5], Hatefi et al.’s method [13], Wang et al.’s method [14], Fei et al.’s method [15], and DS-VIKOR [18]; the experimental results are shown in Table 8. Among them, since DS/AHP, DS-AHP, Hatefi et al.’s method, Wang et al.’s method, Fei et al.’s method, and DS-VIKOR do not specify how to deal with uncertainty evaluation in the form of interval values, we use the method proposed by Ma et al. [4] to generate the corresponding evaluation in the form of the mass function. In addition, since none of these existing methods can handle continuous uncertain evaluations, we replace them with deterministic mathematical expectations.

As can be seen from Table 8, the proposed method and other related methods consistently select *c* as the optimal DA. Moreover, except for the DS-AHP and DS-VIKOR, all other related methods consider the six DAs a,b,c,d,e,h to be relatively better than other DAs, which is consistent with our method.

To further analyze the performance of the priority order generated by our method, we calculated the ranking similarity between the priority order generated by our method and the priority orders generated by other methods. Since the priority order is used to determine the optimal DA, the WS coefficient [44] is used as a measure of ranking similarity in this paper due to its main advantage that differences at the top of the ranking have a more significant impact than differences at the bottom. In addition, to generate our method’s specific priority order, we further compare the two DAs *a* and *b*. We redefine the frame of the mass function for each criterion as $\Theta^{\prime \prime }=\left\{a,b\right\}$ and then repeat Steps 2–3. Then, we obtain the combined final mass function and calculate the plausibility functions of *a* and *b*, yielding Pl(a)=0.701,Pl(b)=0.712, so that b≻a. Therefore, the specific priority order generated by our method is c≻d≻b≻a≻e≻h≻f≻g≻i≻j. Using the priority order generated by our method as the reference ranking, we calculated the WS coefficients of the priority order generated by the other methods with the priority order of our method, as shown in Table 9. As can be seen from Table 9, the WS coefficients of the priority order generated by our method and the priority order of the other methods, except DS-AHP, are all greater than 0.808, above which the similarity between the orderings of ten elements is defined as high [44]. Then, we further check the WS coefficients of the priority order generated by the DS-AHP and other methods, and the results are all less than 0.793, as shown in Table 10. Thus, the WS coefficient of the priority order generated by our method and the priority order generated by the DS-AHP is lower than 0.808 due to the difference between the DS-AHP methods and other methods, while the result generated by our method is consistent with those generated by the majority of the methods. As a result, the rationality of our method can be confirmed.

Moreover, since the mass function of D–S theory has the ability to express ignorance directly, all methods based on D–S theory can easily handle missing discrete evaluations. Furthermore, we compared and analyzed our method with other existing methods in three aspects: whether they can handle continuous evaluation, whether they can handle the discrete evaluation of interval values, and whether they can handle ambiguous discrete evaluation, as shown in Table 11. We can see that only our method performs well in all three aspects, while the other existing MCDM methods based on D–S theory fail to do so. More specifically, among them, Ma et al.’s method is able to deal well with various types of discrete uncertain evaluations, while none of the other methods consider uncertain evaluations in the form of interval values. However, other methods, except ours, cannot handle continuous uncertainty evaluations. As a result, the effectiveness and practicality of our approach are demonstrated.

Next, we analytically compare the numbers of expert knowledge judgments required in the classical AHP method, DS/AHP, DS-AHP, Ma et al.’s method, and our method for an MCDM problem containing *m* DAs with respect to a criterion. This way, our approach’s advantages compared with other AHP-based approaches can be demonstrated.

When using the classical AHP approach, for each criterion, we consider a total of $[m\cdot \left(m-1\right)]/2$ pairwise comparisons, and each pairwise comparison requires the knowledge judgment of the expert. Furthermore, when using the DS/AHP, the expert’s knowledge judgment is required to determine the set of focal elements of the mass function for each criterion, where the focal elements containing more than one DA represent the expert’s ambiguous judgment of the DAs contained within the focal elements. Assuming that the expert determines from their own knowledge judgment that the set of focal elements contains $n_{1}\left(1\leq n_{1}\leq m\right)$ focal elements, it is required that all $n_{1}$ focal elements are compared with the set of DAs, i.e., the frame of discernment. Thus, the total number of knowledge judgments required by the method of DS/AHP consists of two parts, and the first part is the number of knowledge judgments to determine $n_{1}$ focal elements among *m* DAs, which relies on the expert having reasonable knowledge judgments for all *m* DAs. The second part is $n_{1}$ comparative judgments for comparing the focal elements with the frame of discernment. In order to reduce the number of knowledge judgments, the $n_{1}$ obtained by experts based on empirical judgments is much smaller than *m*. Therefore, it can be seen that DS/AHP has a significant reduction in the number of knowledge judgments compared with the classical AHP method. However, it is demanding and laborious for an expert to determine the appropriate set of $n_{1}$ focal elements among *m* DAs with knowledge judgments when the size of *m* is very large, and this method can be very subjective and arbitrary.

When using the DS-AHP method, experts are first required to make knowledge judgments on the performance of *m* DAs. The set of focal elements of the criterion’s mass function is determined by combining all DAs with the same evaluation value into a focal element. Finally, experts are required to make evaluation judgments on each focal element to generate the criterion’s mass function. Assuming that the number of focal elements generated by the DS-AHP method is $n_{2}\left(1\leq n_{2}\leq m\right)$, the number of expert knowledge judgments required by the method consists of two parts: The first part is *m* knowledge judgments for each DA, and the second part is $n_{2}$ knowledge judgments for each focal element. However, identical evaluation values are rare in the practical application of uncertain MCDM, especially for continuous uncertain evaluations. Therefore, in large-scale uncertain MCDM problems, the values of *m* and $n_{2}$ are relatively close. The method of Ma et al. is an extension of DS/AHP. It differs from DS/AHP in generating the mass function for each criterion by requiring an additional knowledge judgment of whether or not the evaluations of the DA groups over the given criterion are complete before assigning a mass value to each focal element.

Our method automatically determines the set of focal elements for each criterion by employing a clustering approach based on uncertain evaluations and then assigning mass values to each focal element by computing the ratios between clusters. Therefore, the number of expert knowledge judgments required by our method is the number of DAs, i.e., *m*, which is significantly less than the number required for the classical AHP, DS/AHP, DS-AHP, and Ma et al.’s method. In addition, our method is efficient in assigning mass values to each focal element, since the ratios between DAs in the same cluster do not need to be calculated, and according to Theorem 1, the ratios between all DAs to be compared can be determined by calculating the ratio of a certain DA to all other DAs. Thus, the efficiency of our method is demonstrated.

## 6. Conclusions

Handling large-scale discrete and continuous uncertainty evaluations of DAs in real-world MCDM applications is an essential and open issue in the research field of MCDM. In order to address this issue, this paper considers the hierarchical structure of the AHP method as the backbone of our MCDM method. Then, the concepts of D–S theory and probability distribution are incorporated to identify various discrete and continuous uncertain evaluations. Afterwards, a clustering technique based on the Silhouette coefficient is applied to translate large-scale uncertain evaluation into the mass value of each criterion. Finally, Dempster’s combination rule is adopted for preference aggregation. Moreover, comparative experiments and analyses with other related methods demonstrate that the proposed method is feasible, effective, and efficient. Our method is considered to be the state of art for the following reasons: First, to the best our knowledge, our method is the first for MCDM problems with large-scale continuous and discrete uncertain evaluations. Hence, the idea of applying the clustering method to the MCDM method explores a new path, and it is able to balance precision and efficiency in handling large-scale decision alternatives. Finally, by choosing the AHP method, one of the most representative and widely used MCDM methods, as the backbone of our method, we indeed extend the application area of the AHP to the handling of real-world MCDM problems with large-scale uncertain evaluations. Moreover, the comparative experiments and analyses with other related methods demonstrate that the proposed method is feasible, effective, and efficient.

In future work, we will apply our method to practical applications to further validate its feasibility. Furthermore, we will integrate methods for determining criteria weights, such as weighting methods that combine the subjective and objective, with our method. Additionally, we will further explore how to avoid the rank reversal paradox. In addition, in this paper, the eigenvector method of the AHP was used to determine the rankings of DAs with respect to each criterion, and since there are many MCDM techniques that can do this, other MCDM techniques can be integrated into our approach to replace the AHP, such as TOPSIS, BWM, etc. Moreover, our method requires pre-determination of the value interval of the optimal number of clusters before clustering, which requires the decision maker to make a basic judgment of the overall DAs to improve the efficiency. Therefore, we can incorporate other clustering methods for uncertain data into our method in the future. Furthermore, extending the D–S theory to handle continuous values would be an exciting topic for future research.

## Figures and Tables

**Table 1:** Decision matrix for the selection of an automotive part supplier.

	Price (Denoted as p, in Dollars/Unit)	Delivery Time (Denoted as t, in Hours)	Service Level (Denoted as sl, in Grades)	Quality (Denoted as q, in Grades)
a	220–230	$P\left(24\leq t\leq 72\right)=\int_{24}^{72}f\left(t\right)dt=1$, where f(t)≈$\left\{\begin{array}{cc} \frac{1}{4\sqrt{2\pi }}e^{-\frac{{\left(x-48\right)}^{2}}{32}}, & 24\leq t\leq 72\\ 0, & \mathrm{otherwise} \end{array}\right.$	VG	70% chance is VP or P, 30% chance is P or A.
b	220–240	24–48	VG	P
c	240	P(t = 12) = 0.2, P(t = 24) = 0.3, P(t = 36) = 0.3, P(t = 48) = 0.2.	VG	A
d	240–245	$P\left(48\leq t\leq 72\right)=\int_{48}^{72}f\left(t\right)dt=1,$ where $f\left(t\right)\approx \left\{\begin{array}{cc} \frac{1}{24}, & 48\leq t\leq 72\\ 0, & \mathrm{otherwise} \end{array}\right.$	VG	A
e	220–250	$P\left(12\leq t\leq 144\right)=\int_{12}^{144}f\left(t\right)dt=1$, where f(t)≈$\left\{\begin{array}{cc} \frac{1}{24}\cdot \exp^{-\frac{1}{24}\left(t-12\right)}, & 12\leq x\leq 144\\ 0, & \mathrm{otherwise} \end{array}\right.$	P	G
f	220–240	130–140	VP-A	null
g	300–350	100–200	A	G
h	390–400	12–72	10% chance is G, 90% chance is VG.	G
i	400–420	null	P or A	20% chance is G or VG, 80% chance is VG.
j	500–600	200–220	G	VG

**Table 2:** Linguistic terms and corresponding numerical assessment grades.

Linguistic Term	Abbreviation	Corresponding Numerical Assessment Grade
Very Poor	VP	1
Poor	P	3
Average	A	5
Good	G	7
Very Good	VG	9

**Table 3:** Converted decision matrix for the selection of an automotive part supplier.

	Price (Denoted as p, in Dollars/Unit)	Delivery Time (Denoted as t, in Hours)	Service Level (Denoted as sl, in Grades)	Quality (Denoted as q, in Grades)
a	$\begin{array}{c} f_{a,p}\left(x\right)=\\ \left\{\begin{array}{cc} \frac{1}{230-220}, & 220\leq x\leq 230\\ 0, & \mathrm{otherwise} \end{array}\right. \end{array}$	$\begin{array}{c} f_{a,t}\left(x\right)=\\ \left\{\begin{array}{cc} \frac{1}{4\sqrt{2\pi }}e^{-\frac{{\left(x-48\right)}^{2}}{32}}, & 24\leq x\leq 72\\ 0, & \mathrm{otherwise} \end{array}\right. \end{array}$	$m_{a,sl}\left(\left\{9\right\}\right)=1.$	$m_{a,q}\left(\left\{1,3\right\}\right)=0.7$, $m_{a,q}\left(\left\{3,5\right\}\right)=0.3$.
b	$\begin{array}{c} f_{b,p}\left(x\right)=\\ \left\{\begin{array}{cc} \frac{1}{240-220}, & 220\leq x\leq 240\\ 0, & \mathrm{otherwise} \end{array}\right. \end{array}$	$\begin{array}{c} f_{b,t}\left(x\right)=\\ \left\{\begin{array}{cc} \frac{1}{48-24}, & 24\leq x\leq 48\\ 0, & \mathrm{otherwise} \end{array}\right. \end{array}$	$m_{b,sl}\left(\left\{9\right\}\right)=1.$	$m_{b,q}\left(\left\{3\right\}\right)=1.$
c	$m_{c,p}\left(240\right)=1.$	$m_{c,t}\left(\left\{12\right\}\right)=0.2,$ $m_{c,t}\left(\left\{24\right\}\right)=0.3,$ $m_{c,t}\left(\left\{36\right\}\right)=0.3,$ $m_{c,t}\left(\left\{48\right\}\right)=0.2.$	$m_{c,sl}\left(\left\{9\right\}\right)=1.$	$m_{c,q}\left(\left\{5\right\}\right)=1.$
d	$\begin{array}{c} f_{d,p}\left(x\right)=\\ \left\{\begin{array}{cc} \frac{1}{245-240}, & 240\leq x\leq 245\\ 0, & \mathrm{otherwise} \end{array}\right. \end{array}$	$\begin{array}{c} f_{d,t}\left(x\right)=\\ \left\{\begin{array}{cc} \frac{1}{24}, & 48\leq x\leq 72\\ 0, & \mathrm{otherwise} \end{array}\right. \end{array}$	$m_{d,sl}\left(\left\{9\right\}\right)=1.$	$m_{e,q}\left(\left\{5\right\}\right)=1.$
e	$\begin{array}{c} f_{e,p}\left(x\right)=\\ \left\{\begin{array}{cc} \frac{1}{250-220}, & 220\leq x\leq 250\\ 0, & \mathrm{otherwise} \end{array}\right. \end{array}$	$\begin{array}{c} f_{e,t}\left(x\right)=\\ \left\{\begin{array}{cc} \frac{1}{24}\cdot \exp^{-\frac{1}{24}\left(t-12\right)}, & 12\leq x\leq 144\\ 0, & \mathrm{otherwise} \end{array}\right. \end{array}$	$m_{e,sl}\left(\left\{3\right\}\right)=1.$	$m_{e,q}\left(\left\{7\right\}\right)=1.$
f	$\begin{array}{c} f_{f,p}\left(x\right)=\\ \left\{\begin{array}{cc} \frac{1}{240-220}, & 220\leq x\leq 240\\ 0, & \mathrm{otherwise} \end{array}\right. \end{array}$	$\begin{array}{c} f_{f,t}\left(x\right)=\\ \left\{\begin{array}{cc} \frac{1}{140-130}, & 130\leq x\leq 140\\ 0, & \mathrm{otherwise} \end{array}\right. \end{array}$	$m_{f,sl}\left(\left\{1,2,3,4,5\right\}\right)$ =1.	$m_{f,q}\left(H\right)=1.$
g	$\begin{array}{c} f_{g,p}\left(x\right)=\\ \left\{\begin{array}{cc} \frac{1}{350-300}, & 300\leq x\leq 350\\ 0, & \mathrm{otherwise} \end{array}\right. \end{array}$	$\begin{array}{c} f_{g,t}\left(x\right)=\\ \left\{\begin{array}{cc} \frac{1}{200-100}, & 100\leq x\leq 200\\ 0, & \mathrm{otherwise} \end{array}\right. \end{array}$	$m_{g,sl}\left(\left\{5\right\}\right)=1.$	$m_{g,q}\left(\left\{7\right\}\right)=1.$
h	$\begin{array}{c} f_{h,p}\left(x\right)=\\ \left\{\begin{array}{cc} \frac{1}{400-390}, & 390\leq x\leq 400\\ 0, & \mathrm{otherwise} \end{array}\right. \end{array}$	$\begin{array}{c} f_{h,t}\left(x\right)=\\ \left\{\begin{array}{cc} \frac{1}{52-12}, & 12\leq x\leq 52\\ 0, & \mathrm{otherwise} \end{array}\right. \end{array}$	$m_{h,sl}\left(\left\{7\right\}\right)=0.1$, $m_{h,sl}\left(\left\{9\right\}\right)=0.9$.	$m_{h,q}\left(\left\{7\right\}\right)=1.$
i	$\begin{array}{c} f_{i,p}\left(x\right)=\\ \left\{\begin{array}{cc} \frac{1}{420-400}, & 400\leq x\leq 420\\ 0, & \mathrm{otherwise} \end{array}\right. \end{array}$	$\begin{array}{c} f_{i,t}\left(x\right)=\\ \left\{\begin{array}{cc} \frac{1}{220-12}, & 12\leq x\leq 220\\ 0, & \mathrm{otherwise} \end{array}\right. \end{array}$	$m_{i,sl}\left(\left\{3,5\right\}\right)=1$.	$m_{i,q}\left(\left\{7,9\right\}\right)=0.2$, $m_{i,q}\left(\left\{9\right\}\right)=0.8$.
j	$\begin{array}{c} f_{j,p}\left(x\right)=\\ \left\{\begin{array}{cc} \frac{1}{600-500}, & 500\leq x\leq 600\\ 0, & \mathrm{otherwise} \end{array}\right. \end{array}$	$\begin{array}{c} f_{j,t}\left(x\right)=\\ \left\{\begin{array}{cc} \frac{1}{220-200}, & 200\leq x\leq 220\\ 0, & \mathrm{otherwise} \end{array}\right. \end{array}$	$m_{j,sl}\left(\left\{7\right\}\right)=1$.	$m_{j,q}\left(\left\{9\right\}\right)=1$.

**Table 4:** Optimal set of clusters of DAs for each criterion from Algorithm 1.

Decision Criteria	Optimal Set of Clusters
price	{a,b,c,d,e,f},{g},{h,i},{j}
delivery time	{a,b,c,d,e,h},{f,g,i},{j}
service level	{a,b,c,d,h},{e,f,i},{g},{j}
quality	{a,b},{c,d,f},{e,g,h},{i,j}

**Table 5:** The mass function of each criterion.

Decision Criteria	Corresponding Mass Function
price	$m_{p}\left(\left\{a,b,c,d,e,f\right\}\right)=0.38$, $m_{p}\left(\left\{g\right\}\right)=0.27$, $m_{p}\left(\left\{h,i\right\}\right)=0.22,$ $m_{p}\left(\left\{j\right\}\right)=0.13.$
delivery time	$m_{t}\left(\left\{a,b,c,d,e,h\right\}\right)=0.69,$ $m_{t}\left(\left\{f,g,i\right\}\right)=0.19,$ $m_{t}\left(\left\{j\right\}\right)=0.12.$
service level	$m_{sl}\left(\left\{a,b,c,d,h\right\}\right)=0.37$, $m_{sl}\left(\left\{e,f,i\right\}\right)=0.13,$ $m_{sl}\left(\left\{g\right\}\right)=0.21,$ $m_{sl}\left(\left\{j\right\}\right)=0.29.$
quality	$m_{q}\left(\left\{a,b\right\}\right)=0.12,$ $m_{q}\left(\left\{c,d,f\right\}\right)=0.20$, $m_{q}\left(\left\{e,g,h\right\}\right)=0.30$, $m_{q}\left(\left\{i,j\right\}\right)=0.38.$

**Table 6:** Mass function of each criterion discounted by the importance weight.

Decision Criteria	Discounted Mass Function
price	${\hat{m}}_{p}\left(\left\{a,b,c,d,e,f\right\}\right)=0.15$, ${\hat{m}}_{p}\left(\left\{g\right\}\right)=0.11$, ${\hat{m}}_{p}\left(\left\{h,i\right\}\right)=0.09,$ ${\hat{m}}_{p}\left(\left\{j\right\}\right)=0.05$, ${\hat{m}}_{p}\left(\Theta \right)=0.6.$
delivery time	${\hat{m}}_{t}\left(\left\{a,b,c,d,e,h\right\}\right)=0.07,$ ${\hat{m}}_{t}\left(\left\{f,g,i\right\}\right)=0.02$, ${\hat{m}}_{t}\left(\left\{j\right\}\right)=0.01$, ${\hat{m}}_{t}\left(\Theta \right)=0.9$.
service level	${\hat{m}}_{sl}\left(\left\{a,b,c,d,h\right\}\right)=0.07,$ ${\hat{m}}_{sl}\left(\left\{e,f,i\right\}\right)=0.03$, ${\hat{m}}_{sl}\left(\left\{g\right\}\right)=0.04$, ${\hat{m}}_{sl}\left(\left\{j\right\}\right)=0.06$, ${\hat{m}}_{sl}\left(\Theta \right)=0.80$.
quality	${\hat{m}}_{q}\left(\left\{a,b\right\}\right)=0.03,$ ${\hat{m}}_{q}\left(\left\{c,d,f\right\}\right)=0.06$, ${\hat{m}}_{q}\left(\left\{e,g,h\right\}\right)=0.09$, ${\hat{m}}_{q}\left(\left\{i,j\right\}\right)=0.12$, ${\hat{m}}_{q}\left(\Theta \right)=0.70$.

**Table 7:** Focal elements and corresponding mass values of $m_{final}$.

{*e*}: 0.015	{*f*}: 0.004	{*g*}: 0.098
{*h*}: 0.021	{*i*}: 0.015	{*j*}: 0.076
{a,b}: 0.028	{c,d}: 0.0066	{e,f}: 0.003
{e,h}: 0.0034	{h,i}: 0.05	{f,i}: 0.0003
{i,j}: 0.057	{c,d,f}: 0.037	{e,g,h}: 0.045
{e,f,i}: 0.012	{f,g,i}: 0.0075	{a,b,c,d}: 0.0088
{a,b,c,d,e}: 0.0067	{a,b,c,d,h}: 0.035	{a,b,c,d,e,f}: 0.089
{a,b,c,d,e,h}: 0.027	Θ: 0.35	

**Table 8:** Comparison of experimental results of existing MCDM methods based on D–S theory.

Method	MCDM Technology Applied	Priority Order	Optimal Choice
DS/AHP [37]	AHP	c≻e≻d≻b ≻h ≻a≻j≻i≻g≻f	c
DS-AHP [27]	AHP	c≻j≻a≻e≻h ≻i≻d≻g≻b≻f	c
Ma et al.’s method [5]	AHP	c≻a≻b≻d ≻e ≻h≻f≻g≻j≻i	c
Hatefi et al.’s method [13]	ARAS	c≻d≻e≻b ≻h ≻a≻j≻g≻i≻f	c
Wang et al.’s method [14]	TOPSIS	c≻e≻h≻d ≻b ≻a≻j≻i≻g≻f	c
Fei et al.’s method [15]	ELECTRE	c≻b,d≻a≻h ≻e≻f,g,i,j	c
DS-VIKOR [18]	VIKOR	c≻e≻d≻b ≻f ≻a≻h≻g≻i≻j	c
Our method	AHP	c≻d≻a,b≻e≻h ≻f≻g≻i≻j	c

**Table 9:** Comparison of the priority order of other existing methods with our method and the corresponding WS coefficients.

Method	*c*	*d*	*b*	*a*	*e*	*h*	*f*	*g*	*i*	*j*	WS Coefficient [44]
DS/AHP [37]	1	3	4	6	2	5	10	9	8	7	0.870
DS-AHP [27]	1	7	9	3	4	5	10	8	6	2	0.722
Ma et al.’s method [5]	1	4	3	2	5	6	7	8	10	9	0.919
Hatefi et al.’s method [13]	1	2	4	6	3	5	10	8	9	7	0.909
Wang et al.’s method [14]	1	4	5	6	2	3	10	9	8	7	0.815
Fei et al.’s method [15]	1	2	2	4	6	5	7	7	7	7	0.954
DS-VIKOR [18]	1	3	4	6	2	7	5	8	9	10	0.873

**Table 10:** The WS coefficients of the priority order generated by the DS-AHP method in comparison with those generated by other methods.

	**DS/AHP [37]**	**Ma et al.’s Method [5]**	**Hatefi et al.’s Method [13]**	**Wang et al.’s Method**
WS coefficient [44]	0.756	0.729	0.763	0.745
	**Fei et al.’s Method** [15]	**DS-VIKOR** [18]	**Our Method**	
WS coefficient [44]	0.793	0.647	0.716	

**Table 11:** Analysis of the comparison of existing MCDM methods based on D–S theory.

	Ability to Handle Continuous Evaluation	Ability to Handle Interval-Valued Discrete Evaluation	Ability to Handle Ambiguous Discrete Evaluation
DS/AHP [37]	No	No	No
DS-AHP [27]	No	No	No
Ma et al.’s method [5]	No	Yes	Yes
Hatefi et al.’s method [13]	No	No	Yes
Wang et al.’s method [14]	No	No	Yes
Fei et al.’s method [15]	No	No	Yes
DS-VIKOR [18]	No	No	Yes
Our method	**Yes**	**Yes**	**Yes**

## Data Availability

Not applicable.

## Abbreviations

The following abbreviations are used in this manuscript:

MCDM	multi-criteria decision making
D–S theory	Dempster–Shafer theory
DA	decision alternative
AHP	analytic hierarchy process
TOPSIS	Technique for Ordering Preference by Similarity to Ideal Solution
VIKOR	VIsekriterijumska optimizacija i KOmpromisno Resenje
ELECTRE	Elimination and Choice Translating Reality
BWM	Best Worst Method
MARCOS	Measurement of Alternatives and Ranking according to Compromise Solution
ARAS	Additive Ratio Assessment
